# Cystathionine γ-lyase S-sulfhydrates SIRT1 to attenuate myocardial death in isoprenaline-induced heart failure

**DOI:** 10.1080/13510002.2023.2174649

**Published:** 2023-02-09

**Authors:** Dan Wu, Yuanyuan Sun, Yijing Gu, Deqiu Zhu

**Affiliations:** a Department of Pharmacy, Tongji Hospital, Tongji University School of Medicine, Shanghai, People’s Republic of China; b Shanghai Engineering Research Center of Organ Repair, School of Medicine, Shanghai University, Shanghai, People’s Republic of China

**Keywords:** Hydrogen sulfide, sirtuin-1, heart failure

## Abstract

**Objective:**

Hydrogen sulfide (H_2_S), the third gasotransmitter, plays a critical role in protecting against heart failure. Sirtuin-1 (SIRT1) is a highly conserved histone deacetylase that has a protective role in the treatment of heart failure by regulating the deacetylation of some functional proteins. This study investigates the interaction between SIRT1 and H_2_S in heart failure and the underlying mechanisms.

**Methods and Results:**

Using endogenous H_2_S-generating enzyme cystathionine γ-lyase (CSE) knockout mice, we found that CSE deficiency aggravated isoprenaline-induced cardiac injury. Treatment with H_2_S attenuated atrial natriuretic peptide level, brain natriuretic peptide level, improved cardiac function. Moreover, H_2_S treatment potentiated myocardial SIRT1 expression. Silencing CSE abolished intracellular SIRT1 expression. Furthermore, CSE/ H_2_S S-sulfhydrated SIRT1 at its zinc finger domains and augmented its zinc ion binding activity to stabilize the alpha-helix structure.

**Discussion:**

In conclusion, these results uncover that a novel mechanism that CSE/H_2_S S-sulfhydrated SIRT1 to prevent heart dysfunction through modulating its activity.

## 1. Introduction

Heart failure is a major global health issue, affecting over 26 million people, but the pathologies of this complex syndrome remain unclear [1,2]. Heart failure may be caused by different etiologies; however, patients with heart failure or heart failure-related disease have high mortality rates and poor quality of life. Statistics showed that the average duration of hospitalization for heart failure patients was 9–10 days, which brings a huge economic burden to society [3]. Mele et al. reported that cardiomyocyte apoptosis was one of the leading causes of heart failure [4]. Therefore, inhibition of cardiomyocyte apoptosis is important to protect against heart failure. Hydrogen sulfide (H_2_S) is the third gaseous mediator like carbon monoxide and nitric oxide, which can regulate cardiovascular functions [5,6]. The endogenous H_2_S is produced by three specific enzymes in mammalian cells: cystathionine β-synthase (CBS), cystathionine γ-lyase (CSE), and 3-mercaptopyruvate sulfurtransferase (3-MST) [5]. Some studies have shown that H_2_S has antioxidant properties [7], anti-atherosclerotic activity [8], pro-angiogenic effects [9], and vascular remodeling effects [10]. The decrease of H_2_S production in the cardiovascular system was observed in CSE knockout (CSE KO) mice, which caused more severe injury in different cardiovascular diseases, including heart failure [11] and myocardial infarction [12]. In contrast, CSE overexpression enhanced the endothelium-dependent vasorelaxation response in the thoracic aorta and abolished the myocardial reperfusion injury [13]. These findings indicate that the endogenous CSE/H_2_S system has a cardiovascular protective effect.

Sirtuin-1 (SIRT1) is a highly conserved nicotinamide adenine dinucleotide (NAD^+^)-dependent class III histone deacetylase that may play a crucial role in heart failure [14,15]. In mammals, SIRT1 is widely distributed in many tissues and organs and protects against apoptosis, oxidative stress, and ischemia/reperfusion injury [16]. Sirt1 mRNA and protein expression were significantly decreased, and the acetylation levels were increased in heart failure [17]. SIRT1 has a modulating effect on many target proteins, such as manganese superoxide dismutase (Mn-SOD), peroxisome proliferator-activated receptor-γ coactivator (PGC-1α), and Forkhead box O (FOXOs) through deacetylation [18]. Previous results suggested that SIRT1 protected against apoptosis through p53 and FoxO1 signaling pathways in cardiomyocytes [19]. H_2_S improved the survival of cardiomyocytes after H_2_O_2_-induced cell death through the SIRT1 signaling pathway [20].

The aim of this study was to determine whether H_2_S could increase SIRT1 activity to protected against heart failure and to unravel its potential role. Moreover, because H_2_S regulated the structure and biological function of the target protein [21] through S-sulfhydration the specific cysteine residue. Therefore, we set out to identify which cysteine residue in SIRT1 that may make a contribution in protective effect of H_2_S.

## 2. Materials and methods

### 2.1. Cell culture and animals

H9c2 cardiomyocytes were purchased from American Type Culture Collection and were cultured in high glucose Dulbecco’s minimum essential medium (DMEM), containing 100 U/ml penicillin–streptomycin, 10% fetal bovine serum (FBS) in 5% CO_2_ at 37°C. H_2_O_2_ (150 μmol/L) was used to induce oxidative stress as described before [20].

Healthy male C57BL/6 (six to eight weeks old) mice were housed in plastic cages and maintained at 23°C and 12 h light/dark cycle (lights on from 7:00 to 19:00). Regular chow and water were available ad libitum. All experimental procedures followed the Shanghai Tongji Hospital Guideline for Animal Use (ethical code: SYXK(Hu) 2019-0005). The CSE knockout (CSE KO) mice (male, six to eight weeks) were a gift from Shanghai Research Center for Model Organisms, Shanghai, China and has been published before [22]. The model of heart failure was induced by subcutaneous injection of isoprenaline (ISO, 7.5 mg/kg) for four weeks once a day as described before [11].

In study-1, wild type (WT) and CSE KO mice were allotted into four groups (*n* = 10 in each group) as below: Group I – WT mice injected subcutaneously with normal saline; Group II – WT mice injected subcutaneously with ISO; Group III – CSE KO mice injected subcutaneously with normal saline; Group IV – CSE KO mice injected subcutaneously with ISO.

In study-2, WT mice were allotted into three groups (*n* = 10 in each group) as below: Group I – injected subcutaneously with normal saline; Group II – injected subcutaneously with ISO; Group III – injected subcutaneously with ISO and injected intraperitoneally with H_2_S donor sodium hydrosulfide (NaHS) (56 μmol/kg/day) as described previously [11].

After four weeks, animals were euthanized with an intraperitoneal injection of pentobarbital (0.1 g/kg), and then hearts as well as serum were harvested for further studies.

### 2.2. Chemicals and antibodies

FBS, DMEM, and 0.25% Trypsin were obtained from Gibco. 100 U/ml penicillin–streptomycin and dimethyl sulfoxide were from Shanghai Yisheng Bio-Technology. Phosphate buffer saline, bicinchoninic acid protein assay kit, radioimmunoprecipitation assay lysis buffer, protease and phosphatase inhibitor cocktail for general use, sodium dodecyl sulfate, ammonium persulfate substitute, Glycine, sodium dodecyl sulfate-polyacrylamide gel electrophoresis loading buffer, 1 M Tris-HCl aminomethane hydrochloride, pH = 6.8, N,N,N′,N′-tetramethylethylenediamine, sodium dodecyl sulfate-polyacrylamide gel electrophoresis loading buffer, 4% paraformaldehyde fix solution, animal RNA isolation kit with spin column, and diethylpyrocarbonate-treated water were from Beyotime Biotech. Hydroxymethyl, paraffin, and non-fat powdered milk were from BBI Life Sciences Corporation. Methanol, tris-buffered saline, ethanol absolute, dimethylbenzene, sodium dodecyl sulfate-polyacrylamide gel electrophoresis loading buffer, and tris (hydroxymethyl) aminomethane hydrochloride (pH = 8.8) were purchased from Sangon Biotech (Shanghai). Thirty percent acrylamide-bisacrylamide (29:1), ponceau staining buffer, and primary antibody dilution buffer were from Beyotime Biotech. Prestained protein marker, horseradish peroxidase-conjugated secondary antibody, protein A/G magnetic beads, HEPES (4-(2-Hydroxyethyl) piperazine-1-ethane sulfonic acid) solution, ethylenediaminetetraacetic acid, S-methyl methanethiosulfonate, HPDP-biotin and west femto maximum sensitivity substrate were from Thermo Fisher Scientific. Anti-caspase 9 antibody (Cat. NO. 9507S), anti-sirtuin-1 antibody (Cat. NO. 8469S), anti-cystathionine gamma-lyase (CSE) antibody (Cat. NO. 19689S), and anti-peroxisome proliferator-activated receptor-γ coactivator (PCG-1α) antibody (Cat. NO. 2178S) were from Cell Signaling Technology. Anti-glyceraldehyde-3-phosphate dehydrogenase (GAPDH) antibody (Cat. NO. 10494-1-AP) and anti-β-actin antibody (Cat. NO. HRP-60008) were from Proteintech. Immunoglobulin G was from Abcam. Heparin sodium injection was purchased from Chengdu Haitong Pharmaceutical. Isoflurane inhalant was purchased from Lunan Better Pharmaceutical. Hematoxylin and eosin staining kit and masson trichrome staining kit were from Beijing Solarbio. The perfect real-time master mix kit was from Takara.

All drugs were obtained from Sigma except for the above drugs.

### 2.3. Western blot

The protein was quantified by the bicinchoninic acid reagent and then mixed with 4× loading buffer at 95°C for 10 min to denature. The target proteins were separated with 10% sodium dodecyl sulfate-polyacrylamide gel electrophoresis and transferred to polyvinylidene fluoride membranes. Ponceau S was used to observe the target band to facilitate subsequent operations, and then use 1× tris-buffered saline with tween-20 to wash the ponceau staining buffer. The membranes were blocked with 5% non-fat milk containing 0.1% tween-20 at room temperature for 1 h and then incubated with primary antibody at 4°C overnight. After washing, chemiluminescence was used to detect the membranes which were incubated with horseradish peroxidase-conjugated secondary antibodies for 1 h at room temperature.

### 2.4. S-sulfhydration assay

The cells homogenized in HEN buffer (containing 1 mmol/L ethylenediaminetetraacetic acid, 250 mmol/L HEPES (4-(2-hydroxyethyl) piperazine-1-ethane sulfonic acid) (pH 7.7) and 0.1 mmol/L neocuproine) were centrifuged at 12,000 r/min for 30 min at 4°C. The proteins were precipitated for 20 min at −20°C, centrifuged, and the supernatant were discarded. The pellet was resuspended in HENS buffer, and then the biotin-HPDP was added to the suspension for 3 h at room temperature. The obtained biotinylated proteins were separated and precipitated by protein A/G magnetic beads, and then the complex was washed with HENS buffer. The proteins were eluted with sodium dodecyl sulfate-polyacrylamide gel electrophoresis sample buffer and analyzed by Western blot.

### 2.5. Echocardiography and cardiac parameters

The echocardiography of mice was performed by Vevo 770 with an RMV 707B high-frequency probe after the last isoproterenol administration. The mice were anesthetized by isoflurane and immobilized on a wooden board in a supine position. The ejection fraction (EF), fraction shortening (FS), left ventricular internal diameter-diastole (LVIDd) and left ventricular end-diastolic volume (LV Vold) were calculated from LV M-mode ultrasonography.

### 2.6. H_2_s concentration analysis

Plasma H_2_S levels were analyzed by reverse high-performance liquid chromatography system. Ten milliliters Tris-HCl (200 mmol/L, pH 8.5) and 70 μl methyl bromide (3.5 mmol/L) were added into the 30 μl lysed myocardial tissue, and then the mixed solution was shaken on a shaker for 1 h at room temperature.

### 2.7. Picrosirius Red staining

The hearts were isolated and fixed in 4% paraformaldehyde solution and then embedded in paraffin. The paraffin-embedded heart sections were cut into 5 µm thickness slices and stained with Picrosirius Red staining solution according to the operation manual. Quantitative histological analysis of myocardial fibrosis was performed using the Image J software.

### 2.8. Plasmid construction

The cDNA of human SIRT1 gene contains 747 amino acids (NCBI number NM_012238.5). The positions of the cysteine residues including C371, C374, C395 and C398 are according to the 747 as human SIRT1 variant (NCBI number NM_012238.5). The primers for site-directed mutagenesis of SIRT1 are as follows:

C371A, forward: 5′-GCCCTGATTTGTAAATACAAAGTTGA-3′

C371A, reverse: 5′-AGATGCTGTTGCAAAGGAACCAT-3′

C374A, forward: 5′-GCTAAATACAAAGTTGACTGTGAAGCTG-3′

C374A, reverse: 5′-AATCAGGCAAGATGCTGTTGCAA-3′

C395A, forward: 5′-GCTCCTAGGTGCCCAGCTGATG-3′

C395A, forward: 5′-TCGAGGAACTACCTGATTAAAAATA-3′

C398A, forward: 5′-GCCCCAGCTGATGAACCGCTT-3′

C398A, forward: 5′-CCTAGGACATCGAGGAACTACCTGATT-3′

### 2.9. Real-time PCR

The total RNA in heart tissue was extracted by the animal RNA Isolation Kit and then the RNA concentration was determined. The reverse transcription kit was used to reverse transcribe the RNA into cDNA. The cDNA was used as a template and then the target genes were amplified by PCR. Glyceraldehyde phosphate dehydrogenase (GAPDH) was used as an internal reference primer for the target genes. The expression of target genes was analyzed by the 2^−ΔΔCT^ method [23]. The Primer sequences: GAPDH forward primer (5′-AATGGATTTGGACGATTGGT-3′) and reverse primer (5′-TTTGCACTGGTACGTGTTGAT-3′), brain natriuretic peptide (BNP) forward primer (5′-GAGGTCACTCCTATCCTCTGG-3′) and reverse primer (5′-GCCATTTCCTCCGACTTTTCTC-3′), atrial natriuretic peptide (ANP) forward primer (5′-GCTTCCAGGCCATATTGGAG-3′), and reverse primer (5′-GGGGGCATGACCTCATCTT-3′).

### 2.10. Statistical analysis

All experimental results are expressed as mean ± SEM. Statistical analyses used GraphPad Prism 7.02 software. Shapiro–Wilk normality test was performed to determine the data distribution. Non-normally distributed data were analyzed by nonparametric tests: Kruskal–Wallis test followed by Dunn’s post hoc analysis for >2 groups or Mann–Whitney test for two groups. Normally distributed data were analyzed by using parametric tests: one-way analysis of variance (ANOVA) followed by Tukey test for >2 groups or unpaired Student’s *t*-test for two groups. Multiple testing adjustment was included in the Dunn’s test and Tukey test through controlling the Type I error for the family of comparisons. Experiments consisting of two categorically independent variables and one dependent variable were analyzed by two-way ANOVA. A *P*-value <.05 was considered statistically significant.

## 3. Results

### 3.1. CSE/H_2_S played a critical role in cardiac function

**Figure 1.**
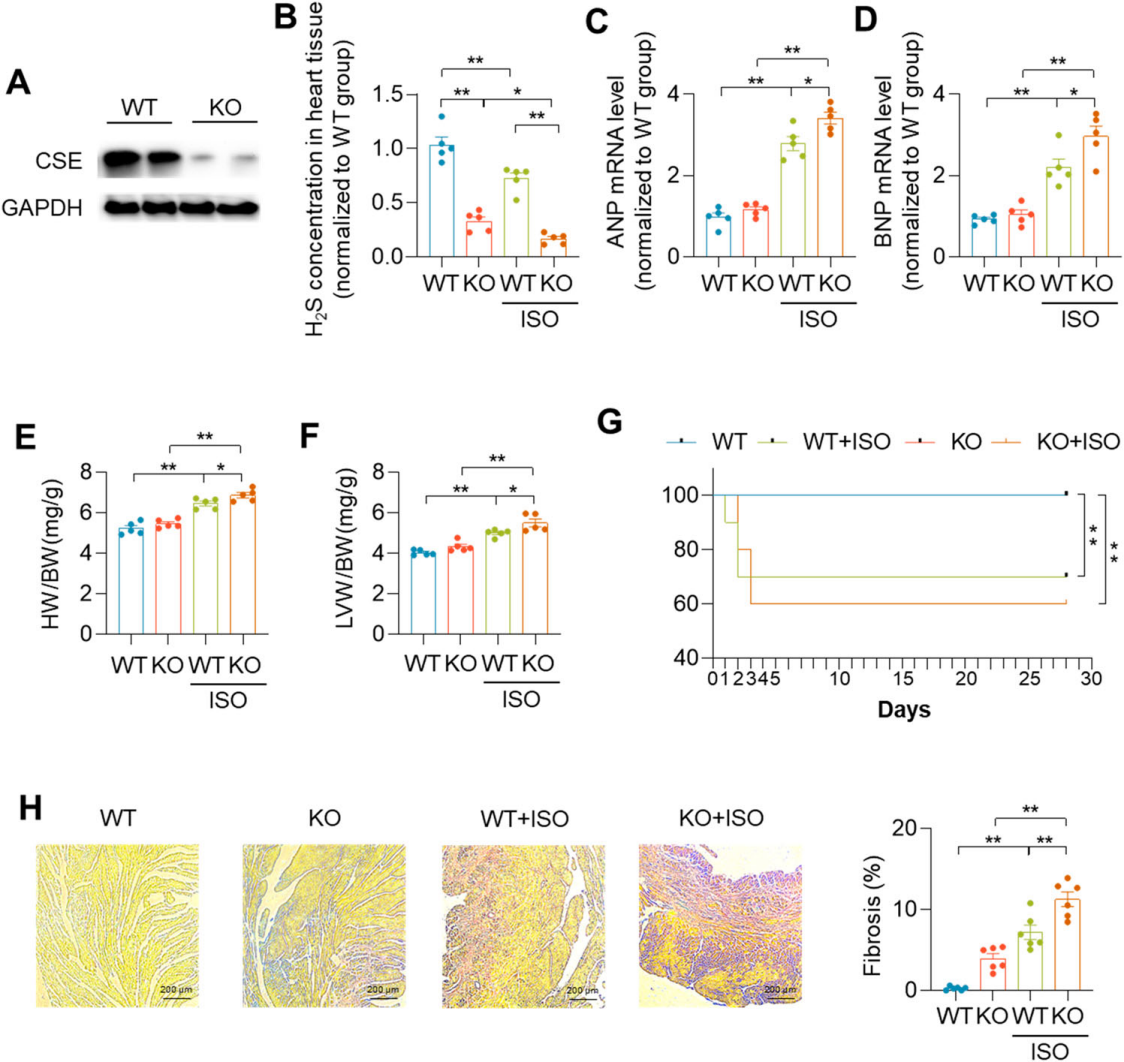
CSE improved ISO-induced heart failure. (A) Western blotting images showing the CSE protein levels in wild type (WT) and CSE knockout (KO) mice heart tissues. (B–D) Quantification of H_2_S concentrations (B), ANP mRNA level (C) and BNP mRNA level (D) in heart tissues. (E, F) Quantification of heart weight/body weight ratio (HW/BW) (E) and left ventricular weight/body weight ratio (LVW/BW) (F). (G) The survival rate in indicated mice. (H) Representative images of Sirius red staining and quantification of fibrosis in indicated mice heart. Scale bar = 200 μm. Data represent means ± SEM, *n* = 5–10. *p < .05, **p < .01.

To investigate whether CSE proteins can regulate cardiac function, we used CSE KO mice to perform experiments. We found that CSE protein expression, as well as H_2_S concentration, were significantly decreased in CSE KO mice compared with WT mice (Figure 1(A,B)). Compared with the control group, ISO significantly increased the levels of ANP mRNA (Accession: AK147180.1) and BNP mRNA (Accession: D82049.1) in the heart, GAPDH mRNA (Accession: AY618568.1) was used as loading control. The treatment of ISO led to higher ratios of left ventricular weight/body weight (LVW/BW) and heart weight/body weight (HW/BW), and increased mortality. Meanwhile, in the ISO-treated group, ANP mRNA levels, BNP mRNA levels, LVW/BW, and HW/BW were increased in CSE KO mice (Figue 1(C–F)). There was no death in WT and CSE KO group. In WT + ISO group, there were one mouse and two mice died in first and second days, respectively. In CSE KO + ISO group, two mice died on the second and third days respectively (Figure 1(G)). Picrosirius Red staining showed an increase of myocardial fibrosis in ISO-treated group, which were severe in CSE KO mice (Figure 1(H)). The above results indicated that CSE protein has a role in regulating cardiac function.

### 3.2. H_2_S protected against the ISO-induced heart failure

**Figure 2.**
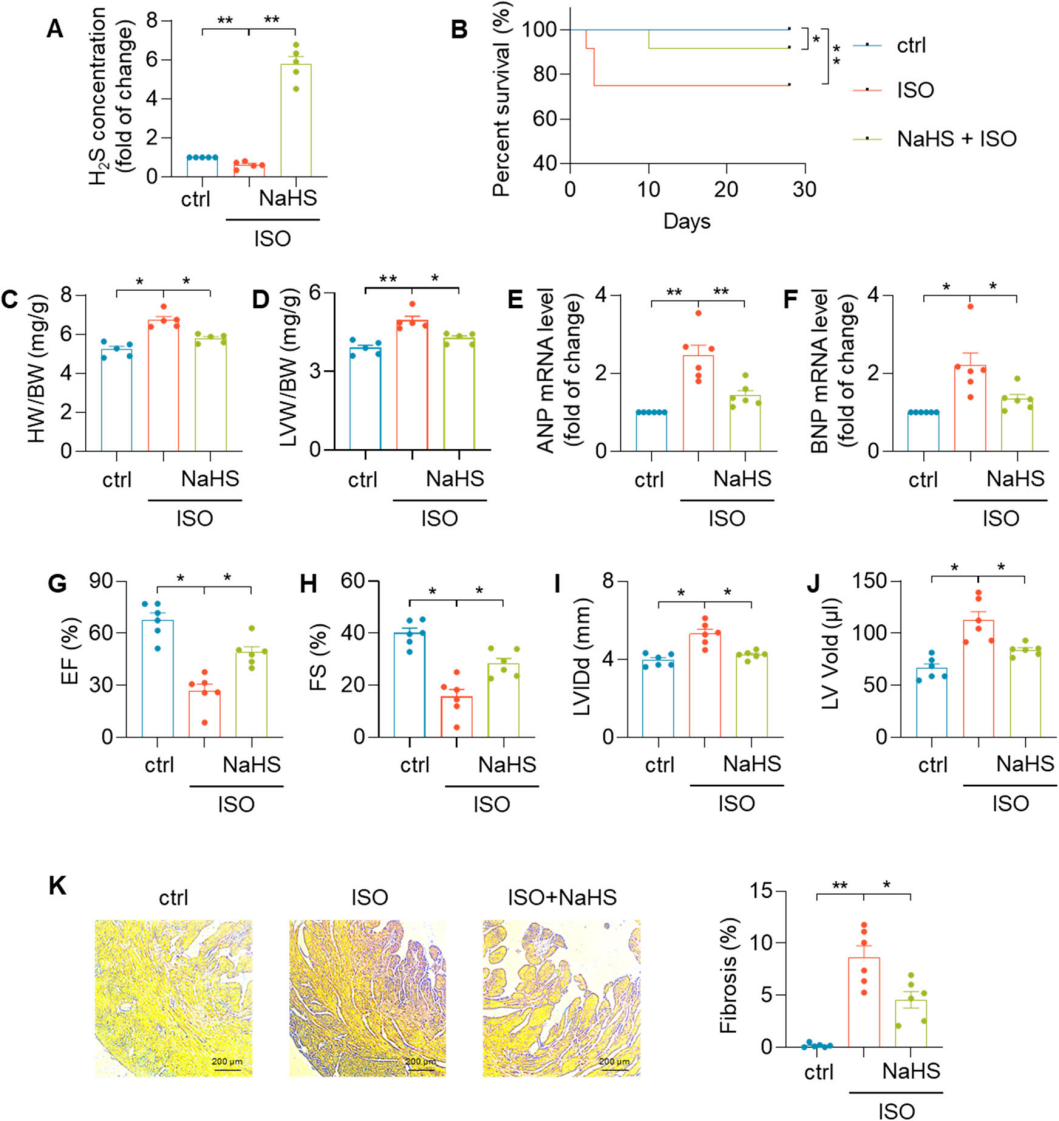
H_2_S protected against ISO-induced heart failure. (A) The survival rate in indicated group. (B) Quantification of H_2_S concentration in heart tissues. (C, D) Heart weight/body weight ratio (HW/BW) (C) and left ventricular weight/body weight ratio (LVW/BW) (D) in indicated group. (E, F) Quantification of ANP mRNA level (E) and BNP mRNA level (F) in heart tissues. (G–J) The echocardiography data showed the changes in ejection fraction (EF), fractional shortening (FS), left ventricular internal dimension diastole (LVIDd), left ventricular end-diastolic volume (LV Vold) in indicated group. (K) Sirius red staining of sections from left ventricular myocardium with indicated treatments for four weeks in mice. Data represent means ± SEM, *n* = 5–10. *p < .05, **p < .01.

NaHS acts as an exogenous H_2_S donor and could increase H_2_S concentration significantly in mouse hearts (Figure 2(A)). As shown by the Kaplan–Meier survival plot (Figure 2(B)), NaHS improved the survival rate of mice in ISO-induced heart failure. The HW/BW and LVW/BW were decreased in the NaHS + ISO group compared with the ISO group (Figure 2(C,D)). Furthermore, treatment of NaHS could inhibit ISO-induced elevation of hypertrophic markers (ANP and BNP) (Figure 2(E,F)). The results of the ultrasonic electrocardiogram indicated that ISO treatment decreased ejection fraction and fractional shortening (Figure 2(G,H)). In contrast, ISO treatment increased left ventricular internal dimension diastole and left ventricular end-diastolic volume (Figure 2(I,J)). ISO treatment also increased myocardial fibrosis (Figure 2(K)). However, NaHS could decrease mortality rate, decrease ANP and BNP mRNA level, improve cardiac function, decrease myocardial fibrosis against ISO-induced abolished myocardial damage caused by ISO. These data suggested that H_2_S was protective against heart failure.

### 3.3. Endogenous CSE/H_2_S activated SIRT1/PGC-1α pathway

**Figure 3.**
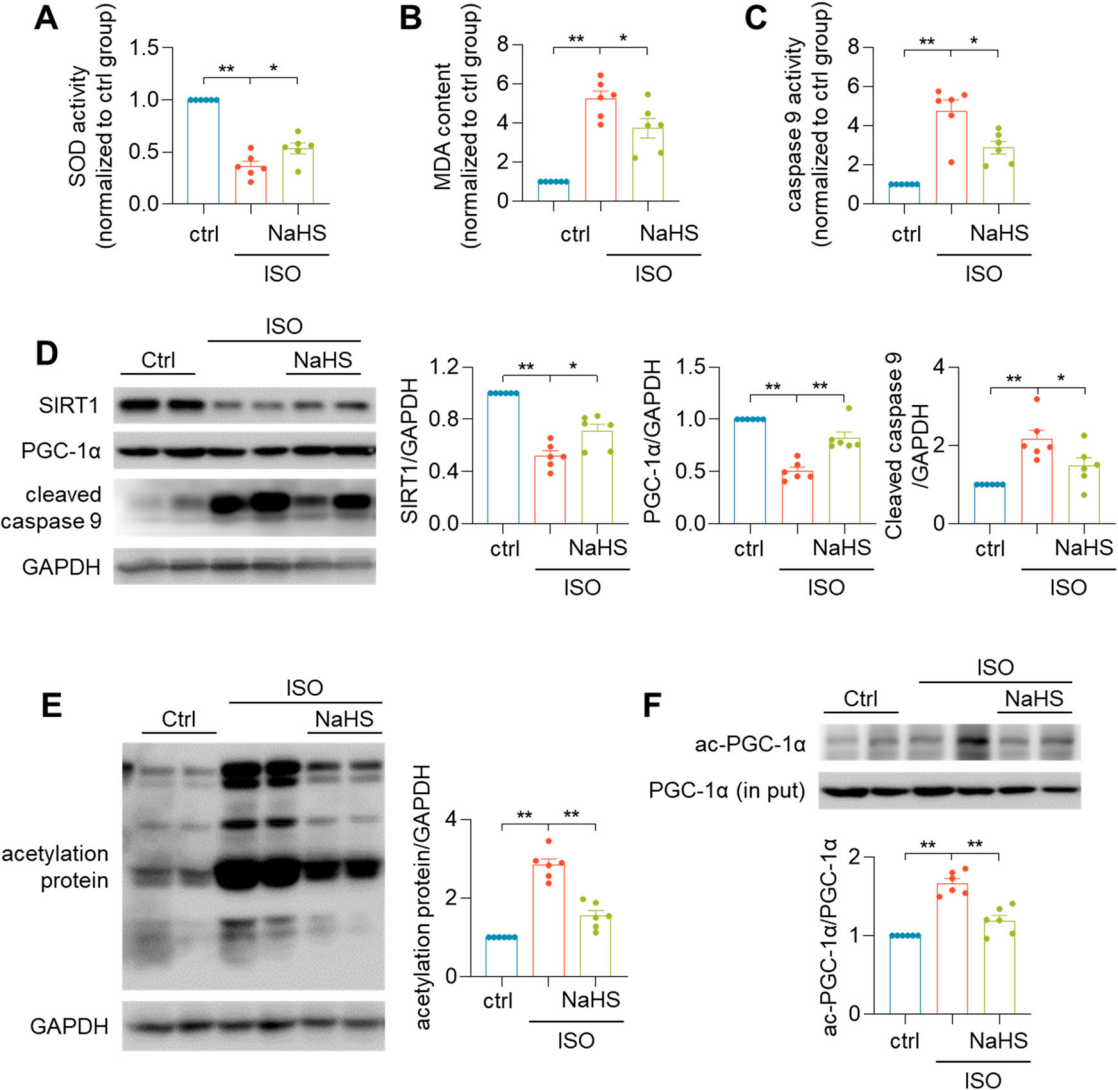
H_2_S increased SIRT1 expression and its activity. (A–C) Quantification of SOD activity (A), MDA content (B) and caspase 9 activity (C) in heart tissues. (D) Western blotting images showing the protein levels of SIRT1 and PGC-1α in indicated heart tissues. (E, F) Western blotting images showing the total acetylation protein level (E) and ac-PGC-1α (F) level in indicated heart tissues. Data represent means ± SEM, *n* = 6. *p < .05, **p < .01.

ISO could induce oxidative stress and apoptosis in heart. Indeed, a decrease activity of the antioxidant enzyme superoxide dismutase (SOD), an increase of malondialdehyde (MDA) content, as well as an increase of caspase 9 activity and cleaved caspase 9 level were noted in ISO-treated heart (Figure 3(A–D)), which could be attenuated by NaHS treatment. The treatment with NaHS significantly upregulated the levels of SIRT1 and PGC-1α in ISO-induced heart failure mice (Figure 3(D)). The protective function of SIRT1 is due to deacetylate its substrates and transcription factors [24]. We found that treatment with ISO increased acetylated protein levels, but NaHS could inhibit the effect of ISO (Figure 3(E)). PGC-1α plays an important role in mitochondrial biogenesis and function [25]. It was reported that PGC-1α was one of the target proteins of SIRT1 [26]. Compared with the ISO group, NaHS significantly decreased the level of acetylated PGC-1α (Figure 3(F)).

**Figure 4.**
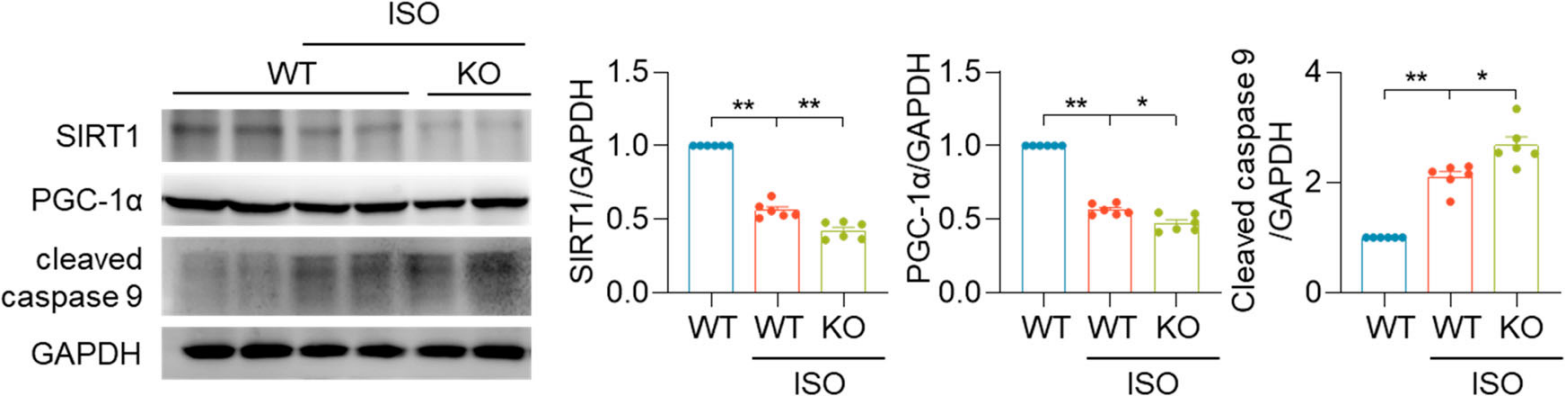
Lack of H_2_S caused severe heart failure. Western blotting images showing the protein levels of SIRT1, PGC-1α and cleaved-caspase 9 protein levels in indicated mice heart tissues. Data represent means ± SEM, *n* = 6. *p < .05, **p < .01.

Upon ISO treatment, SIRT1 and PGC-1α expression levels in CSE KO mice were significantly lower than that in WT mice (Figure 4). Moreover, the cleaved caspase 9 levels in ISO + CSE KO group decreased significantly different compared with the ISO + WT group (Figure 4). These results suggested that the endogenous CSE/H_2_S regulated the deacetylation activity of SIRT1.

### 3.4. H_2_S-sulfhydrated SIRT1 at two CXXC zinc finger domains

**Figure 5.**
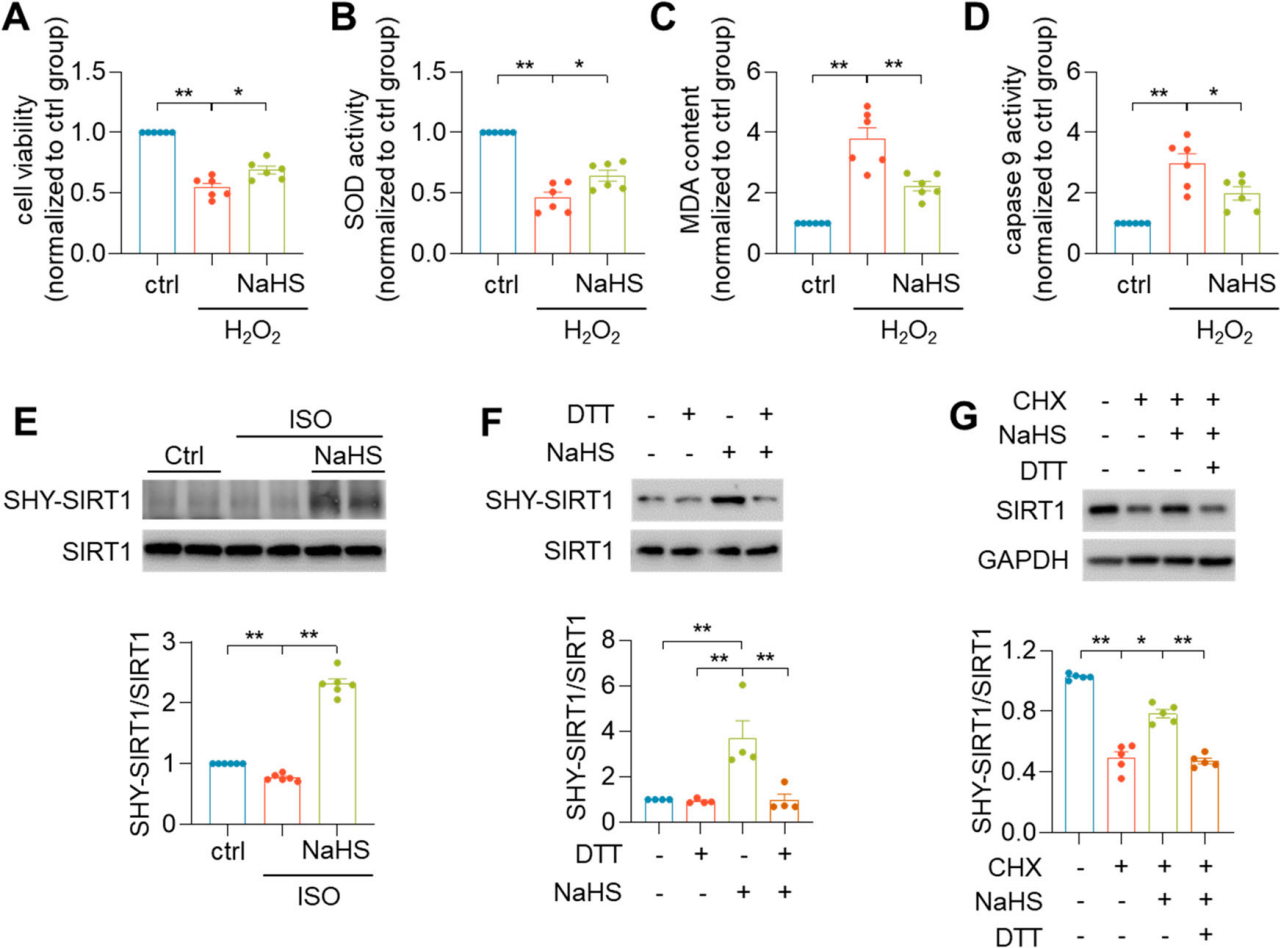
H_2_S protected against oxidative stress through S-sulfhydrated SIRT1. (A–D) Quantification of cell viability (A), SOD activity (B), MDA content (C) and caspase 9 activity (D) in H9c2 cardiomyocytes. (E) Western blotting images showing the levels of SIRT1 S-sulfhydration in indicated hearts tissues. (F) Western blotting images showing the levels of SIRT1 S-sulfhydration upon DTT treatment in H9c2 cardiomyocytes. (G) Western blotting images showing stabilization of SIRT1protein upon CHX treatment in H9c2 cardiomyocytes. Data represent means ± SEM, *n* = 4–6. *p < .05, **p < .01.

Cell viability was reduced in the treatment of H_2_O_2_, which could be increased H_2_S treatment (Figure 5(A)). After H_2_S treatment, increased SOD activity, decreased MDA content and caspase 9 activity were observed in H_2_O_2_-treated H9c2 cardiomyocytes (Figure 5(B–D)). These findings indicated that H_2_S had a marked effect on oxidative stress and apoptosis. To investigate the S-sulfhydration by H_2_S on SIRT1 activity, we determined the expressions of S-sulfhydration of SIRT1 (SHY-SIRT1) by immunoprecipitation-linked biotin switch assay. NaHS increased the expression of SHY-SIRT1 in heart failure (Figure 5(E)). Furthermore, the SHY-SIRT1 was upregulated by NaHS treatment. To further demonstrate SIRT1 was S-sulfhydrated by H_2_S, we used de-sulfhydration reagent dithiothreitol (DTT, 1 mmol/L for 1 h) and found that DTT attenuated SHY-SIRT1 level in the H_2_O_2_-induced H9c2 cardiomyocytes (Figure 5(F)). Cycloheximide (CHX, 25 µg/mL for 24 h) was used to inhibit protein synthesis. NaHS treatment inhibited the effect of CHX on the SIRT1 protein level (Figure 5(G)). Therefore, S-sulfhydration could stabilize SIRT1 and prevent its degradation.

**Figure 6.**
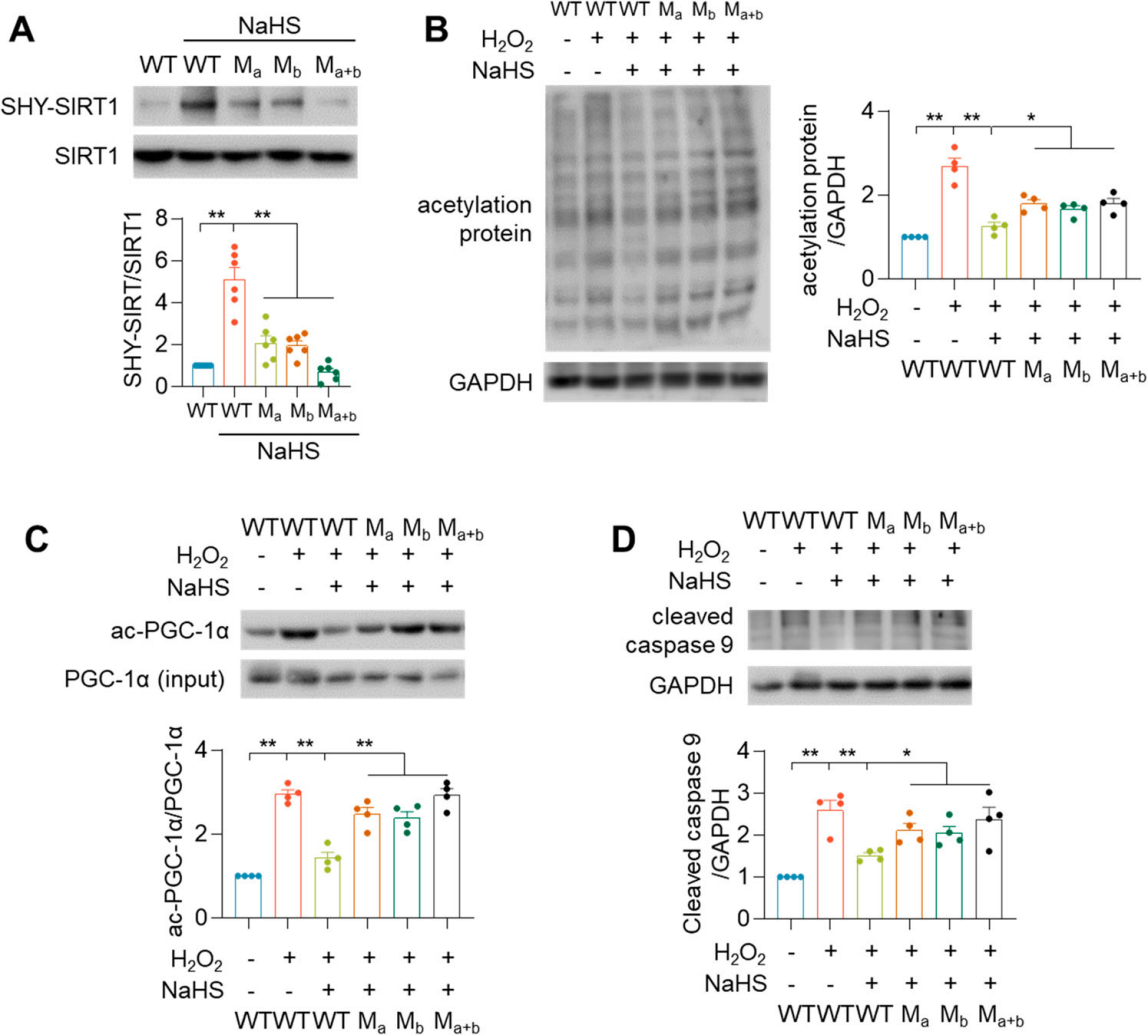
H_2_S S-sulfhydrated SIRT1 at Zinc figure domains. (A) Western blotting images showing the effects of H_2_S on S-sulfhydration of WT or mutations of SIRT1 overexpression in HEK-293 cells. (B–D) Western blotting images showing the effects of H_2_S or/and H_2_O_2_ on total acetylation protein level (B), ac-PGC-1α level (C) and cleaved-caspase 9 expression (D) after WT or mutations of SIRT1 overexpression in HEK-293 cells. Data represent means ± SEM, *n* = 4–6. *p < .05, **p < .01.

To explore the S-sulfhydrated cysteine residue sites, the mutated plasmids of domain a (Ma: C371A and C374A), domain b (Mb: C395A and C398A), or both domains (Ma + b: C371A, C374A, C395A, and C398A) were transfected into HEK-293 cells. We found that the SHY-SIRT1 levels were significantly decreased in the SIRT1 mutation proteins (Figure 6(A)). Furthermore, SIRT1 mutations significantly increased the levels of acetylated protein (Figure 6(B)) and ac-PGC-1α (Figure 6(C)). SIRT1 mutations increased the level of cleaved caspase 9 (Figure 6(D)). SIRT1 has a conserved zinc finger motif, which binds to Zn^2+^ to enhance the structural integrity and deacetylation activity of SIRT1. These data suggested that H_2_S S-sulfhydrated SIRT1 at the two CXXC domains to increase Zn^2+^ binding to SIRT1, augmented the stability of SIRT1, and abrogated oxidative stress-induced degradation of SIRT1.

## 4. Discussion

The growing evidence indicates that H_2_S protected against heart failure, but the mechanism remains still unclear [19]. In this study, we found that the severity of heart failure was much higher in CSE KO mice than in wild-type mice, suggesting that CSE-mediated H_2_S production was important in the cardiac system. Moreover, H_2_S donor could increase SIRT1 expression and its deacetylation activity through S-sulfhydrating at zinc finger domains.

CSE/H_2_S pathway has been proved to play an important role in physiology and pathophysiology [27,28]. CSE KO mice showed a significant decrease in H_2_S bioavailability [29], and worsened cardiac dysfunction and dilatation in heart failure [30]. In our study, the concentration of H_2_S was decreased, and the hypertrophic markers (BNP and ANP) were elevated in CSE KO mice. We also found that H_2_S improved cardiac function and prevented cardiac hypertrophy in ISO-induced heart failure. Endogenous H_2_S production *via* CSE had a cardioprotective effect on heart failure.

It was reported that H_2_S mediated the expression of SIRT1 and its activity [31]. We previously showed that H_2_S increased SIRT1 protein levels to protect against oxidative stress. Moreover, SIRT1 could be regulated by H_2_S to protect against ischemia/reperfusion injury in rats [32]. In this study, the expression of SIRT1 was downregulated by ISO, and NaHS could upregulate its level. However, the SIRT1 level was decreased more in CSE KO mice compared with wild-type mice in ISO-induced heart failure. PGC-1α is an important regulator of mitochondrial biogenesis, which could maintain cellular energy homeostasis [33]. SIRT1 can deacetylation PGC-1α to regulate its activity. The present study showed that H_2_S significantly decreased ac-PGC-1α levels and cleaved caspase-9 in heart failure. However, the levels of ac-PGC-1α and cleaved caspase-9 were abrogated in CSE KO mice as compared to wild-type animals. These results indicated that the CSE/H_2_S upregulated SIRT1 protein level and increased the deacetylation activity of SIRT1. Therefore, H_2_S protected against heart failure via activating SIRT1.

Evidence showed that SIRT1 deacetylated its downstream targets (PGC-1α, p53, FOXOs, etc.) to regulate the oxidative stress, inflammation, and apoptosis process in ischemia/hypoxia injury [34]. Here, we showed that treatment with H_2_S increased the deacetylation activity of SIRT1, but this effect was attenuated by DTT. Therefore, H_2_S improved cardiomyocyte function by S-sulfhydrating SIRT1. SIRT1 protein level and its deacetylase activity were regulated by post-translational modifications such as S-nitrosylation, SUMOylation, phosphorylation, etc. [35] SUMOylation of SIRT1 at Lys 734 increased its deacetylase activity and stabilized its protein [36]. Unlike SUMOylation, SIRT1 was S-nitrosated at Cys 387 and Cys 390 to reduce its deacetylase activity [37]. Du et al. found S-sulfhydration, a novel post-translational modification, at two zinc finger domains of SIRT1 increased SIRT1 deacetylation activity and attenuated atherosclerotic plaque formation [8]. At the same zinc finger domains, SIRT1 activity could be inhibited through S-glutathionylation by GSNO [38]. Zn^2+^ binding to the two conserved CXXC domains of SIRT1 stabilized its alpha-helix structure [39]. In our study, we found H_2_S S-sulfhydrated SIRT1 at Cys371, Cys374, Cys395 and Cys398 sites to increase SIRT1 deacetylation activity in response to oxidative stress. PGC-1α was the master regulator of mitochondrial biogenesis [40]. Acetylation could alter the localization of PGC-1α in the nucleus, and then inhibit PGC-1α transcriptional activity [41]. Multiple studies have shown that the deacetylation of PGC-1α was dependent on SIRT1 activity [42–44]. In this study, the mutation of cysteine residues in the zinc finger domains inhibited S-sulfhydration of SIRT1 and increased ac-PGC-1α and cleaved caspase-9 expression. These data suggested that S-sulfhydration in the zinc finger motif of SIRT1 by H_2_S, elevated the deacetylation of SIRT1 target genes, and then attenuated H_2_O_2_-induced cell apoptosis and oxidative stress.

In summary, we demonstrated that H_2_S directly S-sulfhydrated SIRT1 at zinc figure domains to activate SIRT1 to protective against heart failure treatment with H_2_S may serve as a therapeutic approach to attenuate heart failure development in humans. It should be noted that we cannot preclude that whether additional cysteine residues at SIRT1 could be S-sulfhydrated by H_2_S to regulate cardiac function. And further experiment will hopefully answer these questions.

## Data Availability

The authors confirm that the data supporting the findings of this study are available within the article [and/or] its supplementary materials.
